# Sinus pericranii: a case report and literature review

**DOI:** 10.1016/j.bjorl.2023.101305

**Published:** 2023-08-17

**Authors:** Alan Rodrigues de Almeida Paiva, Eduardo Machado Rossi Monteiro, Tereza Sebastião Nogueira, Matheus Chaves de Oliveira, Pedro Filgueiras de Campos, Rebeca Carolina Campos de Almeida Silva

**Affiliations:** a Hospital Felicio Rocho, Belo Horizonte, MG, Brazil; b Hospital Felicio Rocho, Departamento de Cirurgia do Ouvido, Belo Horizonte, MG, Brazil; c Hermes Pardini, Departamento de Radiologia, Belo Horizonte, MG, Brazil

## Introduction

Sinus Pericranii (SP) is a vascular anomaly, which consists of a transcranial communication between the extracranial venous system and intracranial venous sinuses.^1^ Classic SP is a single vascular dilation in the midline along the superior sagittal sinus, which is its most common location.^1^, ^2^ Nevertheless, the literature has shown presentations that are more and more different from usual.^2^, ^3^ Among the descriptions available, its occurrence in the temporal bone is the rarest among them.^3^ SP is typically asymptomatic and underdiagnosed.

Since SP is a rare vascular anomaly with different presentations, the therapeutic approach and possible complications are still little known.^1^

The objective of this study is to present the case of a patient with SP between the external auditory canal and the sigmoid sinus. The previous history of mastoidectomy points to a traumatic etiology, which is little discussed in the literature.

## Case report

The patient, R.E.S., 65 years old, male, was referred to the otorhinolaryngology outpatient clinic for tumor assessment in the right external auditory canal. The patient had the following clinical manifestations: feeling of discomfort in the external auditory canal and intermittent ipsilateral otorrhagia. This clinical status started after the use of anticoagulant (Rivaroxaban) due to Coronavirus infection.

Regarding his medical history, the patient presented a history of recurrent otitis media associated with otorrhea since childhood in the right ear, but never bleeding. A previous tympanomastoidectomy on the same side was referred to treatment when the patient was 15 years of age, with no intercurrences. As comorbidities, the patient presented with dyslipidemia and colon cancer in the course of chemotherapy treatment.

**Figure 1:**
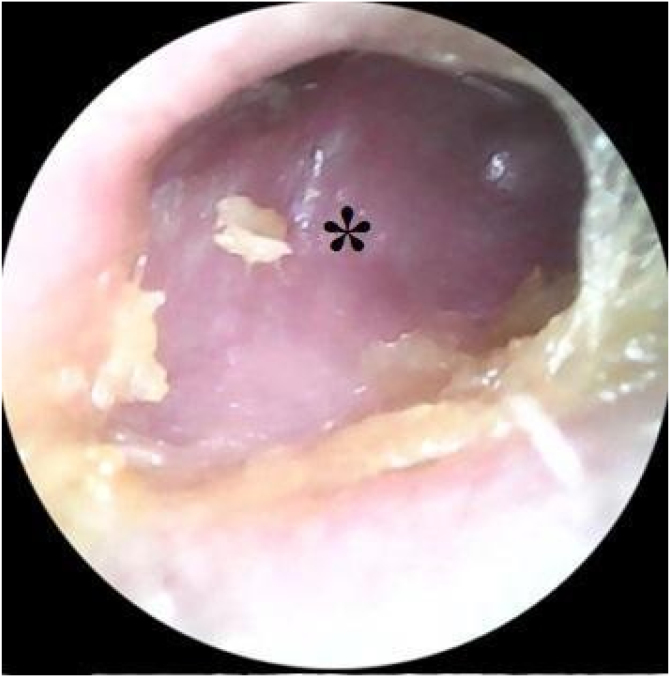
The asterisk shows a bulge in the ear canal.

On physical examination, during otoscopy, bulging of the posterior wall of the right ear canal (Fig. 1) and opacity of the right tympanic membrane with no other changes were seen during otoscopy.

**Figure 2:**
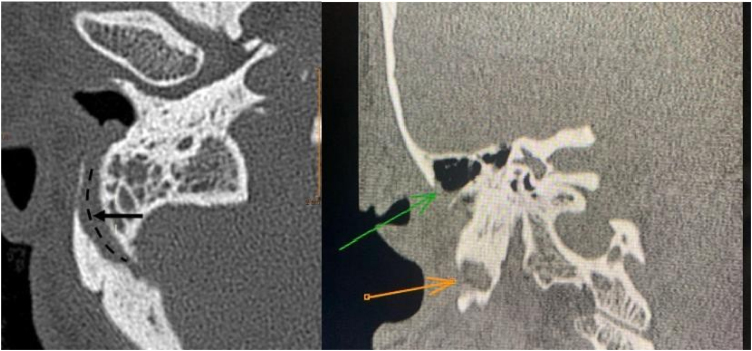
Axial section: the arrow (black) shows the location of the communicating vein, and the dashed line shows its length. Coronal section: orange arrow shows signs of mastoidectomy, and green arrow shows where the lumen of the vessel is.

**Figure 3:**
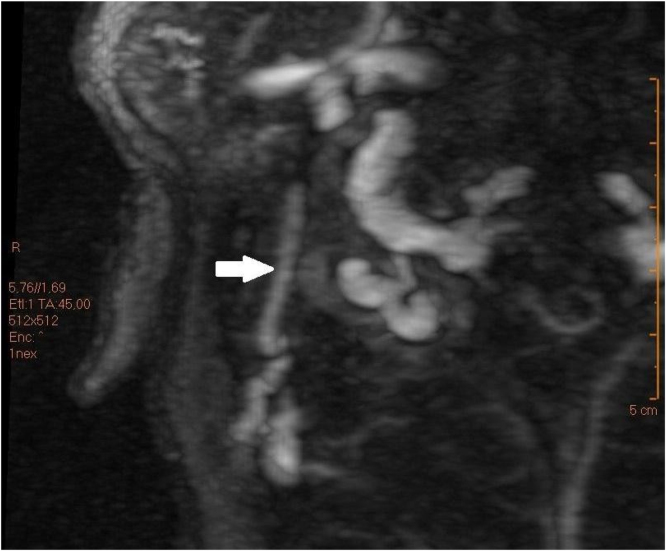
The arrow shows the vein highlighted by a contrast medium.

After assessment, a Computed Tomography (CT) scan of temporal bones was performed (Fig. 2) and had the following findings: signs of mastoidectomy by closed technique, preserving the posterior bony wall of the external auditory canal. Surgical cavity margins the course of the prominent mastoid emissary vein with focuses of exposed lateral cortex with this cortex bordering the surgical cavity. Previously, the referred emissary vein has already been projected towards the posterior and lateral portion of the external auditory canal, with apparent communication between the canal and the sigmoid sinus on the right. After CT, cranial venous magnetic resonance angiography was performed and confirmed sinus pericranii connecting the right external auditory canal to the sigmoid sinus (Fig. 3).

In this case, the option was a conservative treatment and clinical observation. After discontinuation of anticoagulant therapy, the patient evolved with the improvement of the discomfort in the external auditory canal and has not presented new episodes of otorrhagia.

## Discussion

Sinus pericranii consists of a rare vascular anomaly, which involves venous drainage between intracranial and extracranial systems, via cranial diploe, through emissary veins.^1^ The classic presentation of the disease consists of the finding of a non-pulsating soft tissue mass in school-aged individuals.^1^, ^2^ The tumor may be compressed and is emphasized in situations that increase venous pressure. It is mostly asymptomatic and aesthetic problems are usually the reason why patients see a doctor.^2^ When it is symptomatic, the most frequent findings are headache, a feeling of pressure in the head, dizziness and localized pain.^1^, ^2^

Causes of SP are divided into congenital and acquired, however, the etiopathogenesis remains unclear.^1^, ^4^ The most plausible hypotheses for the congenital causes are transient venous hypertension in the final stages of embryogenesis and the absence of venous plexus regression between the periosteum and the dura mater in the closure of cranial sutures.^4^ Among the acquired forms, cranial trauma may cause rupture of emissary veins or of venous sinuses, resulting in the formation of new intra and extracranial connections.^4^

In this case, since there is a neovascularization from surgical trauma, temporal bone surgery seems to be one the causes of SP formation.^1^, ^4^

Its most common location is the frontal region, following the superior sagittal sinus. After that, parietal and occipital regions are the most affected regions.^1^, ^3^ SP in temporal bones is extremely rare with few cases described in the literature, none of them with a direct communication with the external ear, as described in this paper.

The presented report shows atypical and dangerous symptoms of SP, since otorrhagia represents the rupture of the capillary endothelium and blood exposure, with a direct communication of the intracerebral blood flow with the external environment.^1^ Thus, the exposure to ear germs may lead to a severe infection at this site.

Furthermore, with the increasing number of cochlear implants, more cases of SP have been seen in the temporal bone by computed tomography.^5^ Once the SP is identified before surgery, the procedure must be programmed to close the communication or avoid compromising the vessel. In addition, caution should be exercised in outpatient invasive procedures in suspected sinus pericranii.

In the vast majority of cases, treatment is conservative. However, the patient may opt for surgery due to aesthetic deformities or symptomatology.^1^ Some authors have suggested a preventive approach in selected cases due to the risk of bleeding, infections or gas embolism in cases of trauma.^5^

## Conclusion

Sinus pericranii involving the temporal bone is rare and has not been described in the literature as occurring after surgical trauma or direct communication with the external auditory canal.^1^ In this case report, SP connects the sigmoid sinus to the external auditory canal and bulging is seen during otoscopy. Thus, in ear therapeutic approaches, it is necessary to consider the possibility of SP, mainly in patients with previous local surgery. When a decision for surgery is made, it is important to keep in mind that venous malformations not identified by the surgeon or radiologist may have consequences, severe infections, or gas embolism. In this context, although SP is rare, it should be considered in patients who are more susceptible, such as those with congenital syndromes, history of previous trauma or of previous cranial surgery.

## Funding/Support

No funding was secured for this study.

## Conflicts of interest

The authors declare no conflicts of interest.
